# Adult-onset Still’s disease: Sequential atypical rashes—Persistent plaques and flagellate erythema

**DOI:** 10.1016/j.jdcr.2026.08.004

**Published:** 2026-08-17

**Authors:** Katelyn Downey, Adeeb Haroon, Riyad N.H. Seervai, Stephanie J. Mengden, Cyrus H. Faridi, Moshe Y. Bressler, Nicole M. Fett, Jesse J. Keller

**Affiliations:** a Department of Dermatology, Oregon Health & Science University, Portland, Oregon; b Division of Pathology and Laboratory Medicine, Department of Anatomic Pathology, University of Texas MD Anderson Cancer Center, Houston, Texas; c Department of Arthritis and Rheumatology, Oregon Health & Science University, Portland, Oregon

**Keywords:** adult-onset Still’s disease, AOSD, atypical rash, dermatomyositis-like eruption, dyskeratosis, flagellate erythema

Adult-Onset Still’s Disease (AOSD) is a rare systemic inflammatory disease of unknown etiology characterized by high spiking fevers, leukocytosis, arthralgias, and an evanescent salmon-colored rash. Atypical cutaneous presentations, such as persistent purpuric papules or plaques and flagellate erythema, have been recently described, often complicating diagnosis and management.^1^ We present the case of an adult female with AOSD who developed 2 distinct atypical cutaneous morphologies during separate phases of her disease.

## Report of a case

A 30-year-old woman presented with a 6-month history of intermittent high fevers (Tmax 39.4 °C), profound fatigue, 50-pound unintentional weight loss, progressive polyarthralgia, worsening weakness of her upper and lower extremities, and a persistent rash on the neck and lower back for which dermatology was consulted.

**Fig 1:**
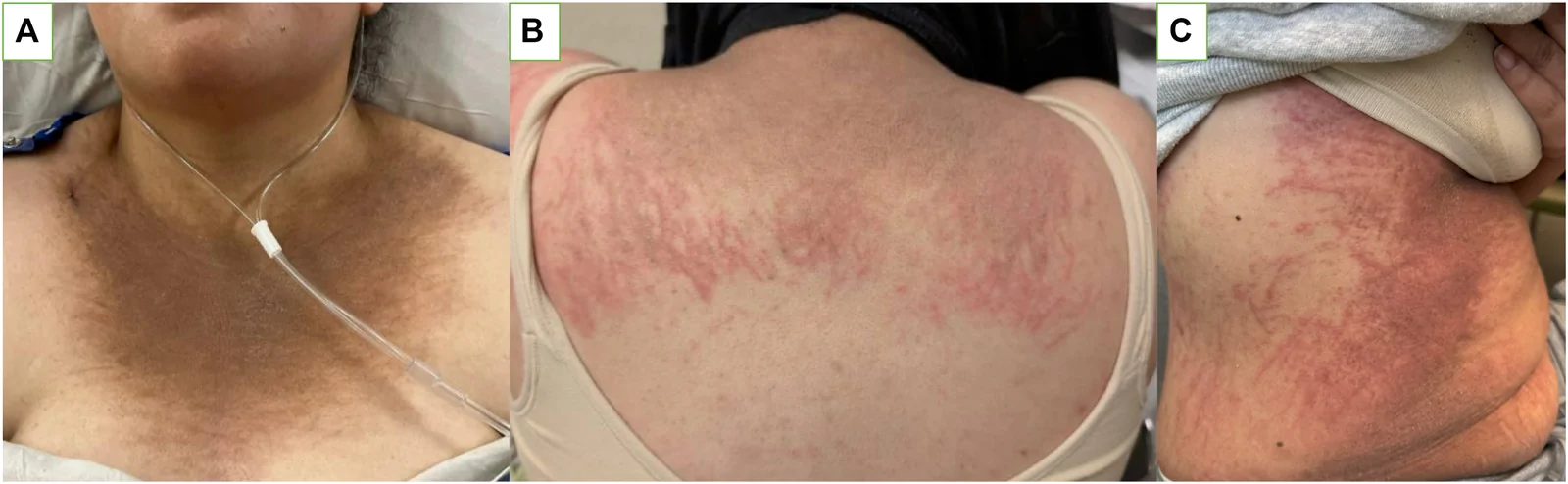
Sequential atypical cutaneous manifestations in adult-onset Still’s disease. **A,** Initial presentation with hyperpigmented, indurated plaques on the anterior neck and upper chest. Subsequent flare demonstrating linear erythematous plaques in a flagellate distribution across the upper back **(B)** and flank **(C)**.

**Fig 2:**
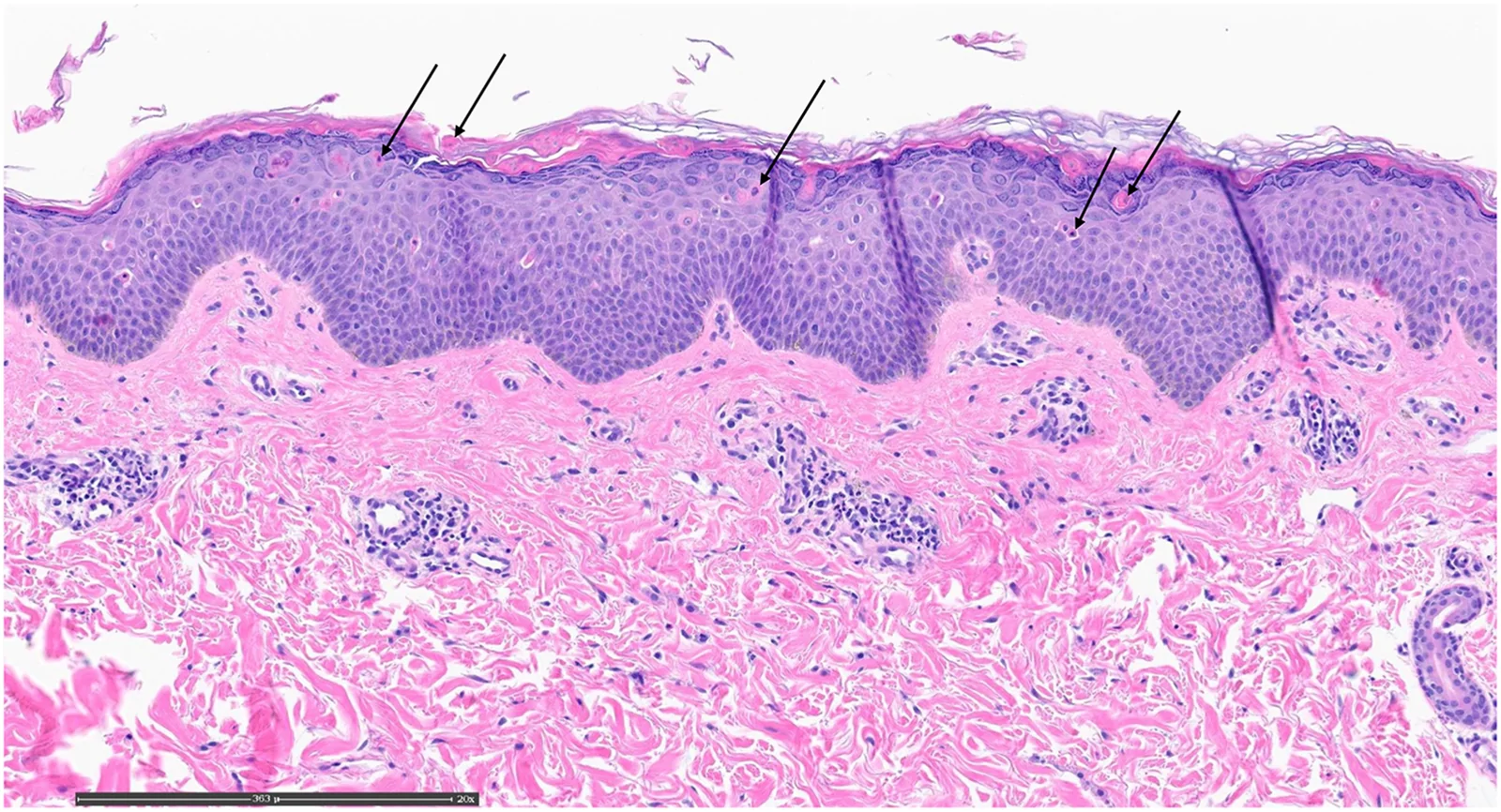
Histopathology of atypical cutaneous lesion in adult-onset Still’s disease Histology shows multiple dyskeratotic keratinocytes (*arrows*) scattered in the upper epidermis, including the stratum corneum, and sparse neutrophils in the papillary dermis, findings characteristic of Still’s disease.

On our exam, she had ill-defined dark pink to brown indurated, papillomatous plaques on the neck, upper chest, central lumbar back, and thighs (Fig 1, *A*). Laboratory studies revealed leukocytosis (20,800 cells/μL with 81.7% neutrophils), anemia, and markedly elevated inflammatory markers (ESR 70-100, CRP 309 mg/L), and ferritin (42,080 ng/mL). Biopsy of the chest demonstrated dyskeratotic cells in the upper epidermis, without accompanying lymphocytes, and a sparse lymphocytic infiltrate with rare neutrophils in the papillary dermis (Fig 2). Subsequent biopsies of non-lesional skin demonstrated a similar pattern of epidermal dyskeratosis in a biopsy of the back, and a sparse purpuric infiltrate with neutrophils on the flank and thigh.

Given her high fevers, polyarthralgia, neutrophilic leukocytosis, markedly elevated ferritin, and elevated inflammatory markers, an extensive infectious, hematologic, and autoimmune evaluation was pursued. Blood cultures were negative, and autoimmune serologies including antinuclear antibody (ANA), rheumatoid factor (RF), anti-cyclic citrullinated peptide (anti-CCP), anti-Smith/RNP, and a myositis-specific antibody panel (including anti-SAE1, anti-NXP2, anti-MDA5, and anti-TIF1-γ antibodies) were negative. Following exclusion of alternative infectious, autoimmune, and malignant etiologies, the patient fulfilled the Yamaguchi classification criteria supporting the diagnosis of AOSD.^2^ She was treated with intravenous methylprednisolone 40 mg daily and subcutaneous anakinra 100 mg daily, resulting in rapid improvement in systemic symptoms and her rash. She was discharged on prednisone 30 mg daily with a taper of 10 mg per week but subsequently developed pruritic injection-site reactions to anakinra, prompting discontinuation approximately 2 months after initiation.

Three days after discontinuing anakinra, she experienced recurrence of fevers, polyarthralgia, and a new rash. On re-admission, dermatologic exam showed linear erythematous thin plaques in a flagellate distribution across the neck, shoulders, flank, and abdomen (Fig 1, *B* and *C*). No new medications, exposures, or alternative etiologies for flagellate dermatitis were identified. She was transitioned to subcutaneous canakinumab 300 mg every 4 weeks with improvement in systemic symptoms and gradual resolution of the rash.

## Discussion

This case highlights the polymorphic nature of AOSD and the significant diagnostic challenges posed by atypical cutaneous manifestations. While the patient met Yamaguchi criteria based on classic systemic features, her skin presentation deviated significantly from the classic evanescent, salmon-pink rash. The patient’s initial presentation of persistent, indurated plaques in a photo-distributed pattern with associated proximal muscle weakness closely mimicked dermatomyositis (DM), a known differential diagnosis in AOSD.^3^ Despite MRI findings suggestive of a nonspecific myopathy, normal creatine kinase, negative myositis-specific antibodies, and a muscle biopsy without evidence of myositis supported exclusion of DM. The resulting diagnostic uncertainty contributed to the 6-month diagnostic delay.

There are increasing reports describing the atypical cutaneous manifestations of AOSD which include persistent pruritic papules and plaques, DM-like eruptions, flagellate erythema, urticarial or purpuric eruptions, and linear or Koebnerized lesions.^1^ Several reports also describe patients developing multiple atypical morphologies either simultaneously or sequentially during the course of disease, reflecting the polymorphic cutaneous spectrum of AOSD. Previously reported cases of simultaneous or sequential AOSD atypical cutaneous manifestations are summarized in Table I.

**Table I:** Reported cases of simultaneous or sequential atypical cutaneous manifestations in adult-onset Still’s disease

Reference	Typical rash	Atypical morphology	Histology	Timing
Santa 2017 (Case 2)^4^	None	Facial edema/erythema; hyperpigmented patches; Flagellate erythema	Upper epidermal dyskeratosis; sparse perivascular lymphocytes/neutrophils	Simultaneous
Qiao 2019 (Case 1)^5^	None	Dermatomyositis-like pruritic plaques; Koebnerization	Upper epidermal dyskeratosis; superficial lymphocytes/neutrophils	Simultaneous
Qiao 2019 (Case 2)^5^	None	Dermatomyositis -like pruritic plaques; Koebnerization	Upper epidermal dyskeratosis; superficial neutrophils	Simultaneous
Tseng 2014^6^	None	Morbilliform pruritic patches → urticaria	Not reported	Subsequent
Michailidou 2015^7^	Classic rash present	Erythematous plaques; linear urticarial eruption; Persistent pigmented plaque	Dermal perivascular inflammatory infiltrate	Subsequent
Affleck 2005^8^	None	Migratory urticated plaques → fixed hyperpigmented plaques	Necrotic keratinocytes; eosinophilic dermal infiltrate	Subsequent
Fernández Camporro 2022 (Case 1)^9^	None	Periorbital erythema; flagellate plaques; violaceous papules	Upper epidermal dyskeratosis; eosinophilic infiltrate	Simultaneous
Fernández Camporro 2022 (Case 2)^9^	None	Periorbital violaceous macules; flagellate papules/plaques	Upper epidermal dyskeratosis; eosinophilic infiltrate	Simultaneous
Qiao 2016^10^	None	Persistent papules/plaques; Koebnerization	Epidermal dyskeratosis; neutrophilic perivascular infiltrate	Simultaneous
Kikuchi 2014 (Case 2)^11^	None	Persistent papules/plaques; linear streaks/pigmentation	Necrotic/apoptotic keratinocytes	Simultaneous
Kikuchi 2014 (Case 3)^11^	None	Persistent papules/plaques; linear erythema	Epidermal dyskeratosis	Simultaneous
Kikuchi 2014 (Case 6)^11^	None	Persistent plaques; urticarial erythema	Corneal layer dyskeratosis	Simultaneous
Omigawa 2018^12^	Evanescent macules	Linear macules → purpura	Epidermal dyskeratosis; vacuolar change; perivascular infiltrate	Subsequent
Liu 2018 (Case 2)^13^	None	Persistent pruritic eruption; edematous erythema with linear configuration	Epidermal dyskeratosis; superficial inflammatory infiltrate	Simultaneous
Pawar 2020^14^	None	Persistent papules/plaques; flagellate erythema	Necrotic keratinocytes; perivascular/periadnexal infiltrate	Simultaneous
Bhatia 2019^15^	None	Persistent erythema; periorbital edema; flagellate erythema	Mild spongiosis; neutrophilic dermal infiltrate	Simultaneous
Prendiville 2004 (Case 1)^16^	Fever-associated rash	Persistent papules/plaques; linear urticarial eruption; periorbital edema; recurrent urticaria	Not reported	Subsequent
Prendiville 2004 (Case 2)^16^	None	Linear urticarial wheals; periorbital edema	Not reported	Simultaneous
Prendiville 2004 (Case 3)^16^	Fever-associated rash	Urticarial eruption; linear streaks; periorbital edema	Not reported	Simultaneous
Prendiville 2004 (Case 4)^16^	None	Urticarial eruption; linear lesions; periorbital edema	Not reported	Simultaneous
Prendiville 2004 (Case 5)^16^	None	Urticaria → persistent papules/plaques with linear morphology	Not reported	Subsequent
Fujii 2003 (Case 1)^17^	Typical rash initially	Persistent erythema → edematous linear plaques	Dermal mononuclear infiltrate with neutrophils	Subsequent
Fujii 2003 (Case 2)^17^	None	Urticarial erythema → persistent generalized erythema	Dermal mononuclear infiltrate with neutrophils	Subsequent
Moraites 2012^18^	None	Lacy dermatitis; dermatographism → heliotrope-like manifestation	Mild perivascular neutrophilic infiltrate	Subsequent
Riyaz 2015^19^	None	Persistent plaques; Koebnerization; flagellate erythema	Basal degeneration; neutrophilic perivascular infiltrate	Simultaneous
Ciliberto 2013^20^	No	Persistent papules/plaques; flagellate erythema	Orthokeratosis; clustered upper epidermal dyskeratosis	Simultaneous
Suzuki 2001^21^	Evanescent rash initially	Persistent plaques; linear pigmentation	Mild perivascular infiltrate; epidermal dyskeratosis	Simultaneous
Nassereddine 2018 (Case 1)^22^	Recurrent urticaria	Persistent hyperpigmented plaques; urticaria; Koebnerization	Hyperkeratosis; dyskeratotic keratinocytes; polymorphous dermal infiltrate	Simultaneous

However, these morphologies are not specific to AOSD and should prompt consideration of a broad differential diagnosis. In particular, persistent papules and plaques may resemble DM, connective tissue diseases such as cutaneous lupus erythematosus, drug eruptions, or malignancy, whereas flagellate erythema should also prompt consideration of bleomycin exposure, shiitake mushroom dermatitis, DM, and other inflammatory dermatoses (Table II).^23^^,^^24^ Because AOSD remains a diagnosis of exclusion, infection, autoimmune, and malignant etiologies should be carefully evaluated before establishing the diagnosis. This principle extends beyond the initial diagnosis. In patients with established AOSD who develop new cutaneous findings while receiving immunosuppressive therapy, clinicians should consider alternative explanations, including drug reactions and infectious complications, rather than attributing all skin findings to disease activity.

**Table II:** Differential diagnoses considered for the atypical cutaneous findings in our patient with adult-onset Still’s disease

Cutaneous morphology	Differential diagnosis	Distinguishing features
Persistent papules and plaques^23^	Dermatomyositis	Gottron papules, heliotrope eruption, shawl or V-sign distribution, muscle weakness, elevated muscle enzymes, or myositis-specific antibodies
	Cutaneous lupus erythematous	Photosensitivity, lupus-specific morphology, interface dermatitis with dermal mucin on histology, or positive ANA and/or lupus-specific antibodies
	Drug eruption	Temporal association with medication exposure; eosinophils may be present on histology
	Cutaneous T-cell lymphoma	Progressive or treatment-refractory patches and/or plaques; clonal proliferation of atypical lymphocytes exhibiting epidermotropism and/or folliculotropism on histology
Flagellate Erythema^24^	Bleomycin or peplomycin exposure	Relevant medication exposure; subsequent linear hyperpigmentation may occur
	Shiitake mushroom dermatitis	Recent ingestion of raw or undercooked shiitake mushrooms; intensely pruritic eruption
	Dermatomyositis	As above

In this case, the initial biopsy revealed superficial dyskeratosis, a key diagnostic clue in this atypical presentation. Unlike the classic evanescent rash, which typically shows only a sparse perivascular infiltrate lacking epidermal change, superficial dyskeratosis is a recognized feature of atypical AOSD.^25^ Its presence served as a pathologic anchor leading to the diagnosis despite the clinically misleading plaque morphology.

This patient’s response to both anakinra and canakinumab supports the central role of the IL-1 pathway in driving the systemic inflammation and the refractory, sequential cutaneous manifestations of AOSD. It is important for clinicians to recognize that AOSD can manifest with multiple, non-evanescent eruptions, and atypical morphologies may accompany more severe or prolonged systemic inflammation.^26^ Recognition of these atypical forms is essential for timely diagnosis and initiation of IL-1 directed therapy.

## Key teaching points

o Adult-onset Still’s disease may present with persistent, atypical cutaneous eruptions, rather than the classic evanescent rash.
o Atypical morphologies include persistent papules and plaques, dermatomyositis-like eruptions, and flagellate erythema.
o Superficial epidermal dyskeratosis on biopsy is an important histopathologic clue in atypical AOSD.
o Recognition of these atypical patterns can reduce diagnostic delay and facilitate timely IL-1 directed therapy.

## Conflict of interest

Fett serves on the Medical Dermatology Society (MDS) Education Committee, is an author/editor for UpToDate, an investigator for AstraZeneca, and an advisory board member for Priovant. Keller is a speaker for Galderma. Seervai is a member of the Derm In-Review Advisory Council affiliated with SanovaWorks/WebMD. Downey, Haroon, Mengden, Faridi, and Bressler have no disclosures.

## References

[1] Calabrese L., D'Onghia M., Cartocci A. (2025). Unfolding dermatological spectrum of Still's disease: a cohort study from the International AIDA Network Still's Disease Registry. Rheumatology (Oxford).

[2] Yamaguchi M., Ohta A., Tsunematsu T. (1992). Preliminary criteria for classification of adult Still's disease. J Rheumatol.

[3] Segovia Rodríguez I., Vicente de la Sota J., Hernández Piriz A. (2025). Adult-Onset still's disease with dermatomyositis-like lesions. Eur J Case Rep Intern Med.

[4] Santa E., McFalls J.M., Sahu J., Lee J.B. (2017). Clinical and histopathological features of cutaneous manifestations of adult-onset still disease. J Cutan Pathol.

[5] Qiao J., Pan Y., Li S. (2019). Adult-Onset still disease presenting with dermatomyositis-like persistent pruritic lesions. Am J Dermatopathol.

[6] Tseng H.C., Lee C.H. (2014). Refractory urticaria in adult-onset still's disease. Rheumatol Int.

[7] Michailidou D., Shin J., Forde I., Gopalratnam K., Cohen P., DeGirolamo A. (2015). Typical evanescent and atypical persistent polymorphic cutaneous rash in an adult Brazilian with Still's disease: a case report and review of the literature. Auto Immun Highlights.

[8] Affleck A.G., Littlewood S.M. (2005). Adult-onset still's disease with atypical cutaneous features. J Eur Acad Dermatol Venereol.

[9] Fernández Camporro Á., Rodriguez Diaz E., Beteta Gorriti V., Gonzalvo Rodríguez P., Alvarez-Cuesta C. (2022). Still disease with persistent atypical dermatomyositis-like skin eruption: two cases associated with macrophage activation syndrome. Clin Exp Dermatol.

[10] Qiao J., Bai J., Fang H. (2016). Persistent pruritic lesions in adult-onset still's disease. Am J Med Sci.

[11] Kikuchi N., Satoh M., Ohtsuka M., Yamamoto T. (2014). Persistent pruritic papules and plaques associated with adult-onset still's disease: report of six cases. J Dermatol.

[12] Omigawa C., Hashimoto T., Hanafusa T., Namiki T., Igawa K., Yokozeki H. (2018). Generalized Purpura as an atypical skin manifestation of adult-onset still's disease in a patient with Behçet’s disease. Acta Derm Venereol.

[13] Liu Y., Ikawa T., Tada Y. (2018). Two severe cases of adult-onset still's disease with persistent pruritic eruptions. Acta Dermato-Venereologica.

[14] Pawar M., Zawar V., Kumavat S. (2020). Persistent pruritic papules AND plaques and flagellate erythema as presenting manifestations of an adult onset still's disease. Actas Dermo-Sifiliográficas (English Edition).

[15] Bhatia R., Kaeley N., Kant R., Basi A. (2019). Fever with persistent flagellate erythema in a primigravida: a rare presentation of adult-onset still's disease. BMJ Case Rep.

[16] Prendiville J.S., Tucker L.B., Cabral D.A., Crawford R.I. (2004). A pruritic linear urticarial rash, fever, and systemic inflammatory disease in five adolescents: adult-onset still disease or systemic juvenile idiopathic arthritis sine arthritis?. Pediatr Dermatol.

[17] Fujii K., Konishi K., Kanno Y., Ohgou N. (2003). Persistent generalized erythema in adult-onset still's disease. Int J Dermatol.

[18] Moraites E., Myers D.J., Lloyd R., Longley B.J. (2012). A 38-Year-Old woman with eyelid Discoloration—Diagnosis. Arch Dermatol.

[19] Riyaz N., Manikath N., Sasidharanpillai S., Govindan A., Davul H., Rini M.R. (2015). Persistent pruritic rash: a rare manifestation and possible poor prognostic sign in adult onset still's disease. Indian J Dermatol Venereol Leprol.

[20] Ciliberto H., Kumar M.G., Musiek A. (2013). Flagellate erythema in a patient with fever. JAMA Dermatol.

[21] Suzuki K., Kimura Y., Aoki M. (2001). Persistent plaques and linear pigmentation in adult-onset still's disease. Dermatology.

[22] Nassereddine H., Fite C., Kottler D. (2018). An atypical persistent eruption of adult-onset still's disease with neutrophilic urticarial dermatosis-like dermal features: a case report and review of the literature. J Cutan Pathol.

[23] Sun N.Z., Brezinski E.A., Berliner J. (2015). Updates in adult-onset still disease: atypical cutaneous manifestations and associations with delayed malignancy. J Am Acad Dermatol.

[24] Yamamoto T., Nishioka K. (2006). Flagellate erythema. Int J Dermatol.

[25] Fortna R.R., Gudjonsson J.E., Seidel G. (2010). Persistent pruritic papules and plaques: a characteristic histopathologic presentation seen in a subset of patients with adult-onset and juvenile Still's disease. J Cutan Pathol.

[26] Narváez Garcia F.J., Pascual M., López de Recalde M. (2017). Adult-onset still's disease with atypical cutaneous manifestations. Medicine (Baltimore).

